# Construction and effectiveness evaluation of a knowledge-based infectious disease monitoring and decision support system

**DOI:** 10.1038/s41598-023-39931-8

**Published:** 2023-08-14

**Authors:** Mengying Wang, Mo Jia, Zhenhao Wei, Wei Wang, Yafei Shang, Hong Ji

**Affiliations:** 1 Information Management and Big Data Center, Peking University Third Hospital, Beijing, China; 2 Goodwill Hessian Health Technology Co. Ltd, Beijing, China

**Keywords:** Data processing, Software

## Abstract

To improve the hospital's ability to proactively detect infectious diseases, a knowledge-based infectious disease monitoring and decision support system was established based on real medical records and knowledge rules. The effectiveness of the system was evaluated using interrupted time series analysis. In the system, a monitoring and alert rule library for infectious diseases was generated by combining infectious disease diagnosis guidelines with literature and a real medical record knowledge map. The system was integrated with the electronic medical record system, and doctors were provided with various types of real-time warning prompts when writing medical records. The effectiveness of the system's alerts was analyzed from the perspectives of false positive rates, rule accuracy, alert effectiveness, and missed case rates using interrupted time series analysis. Over a period of 12 months, the system analyzed 4,497,091 medical records, triggering a total of 12,027 monitoring alerts. Of these, 98.43% were clinically effective, while 1.56% were invalid alerts, mainly owing to the relatively rough rules generated by the guidelines leading to several false alarms. In addition, the effectiveness of the system's alerts, distribution of diagnosis times, and reporting efficiency of doctors were analyzed. 89.26% of infectious disease cases could be confirmed and reported by doctors within 5 min of receiving the alert, and 77.6% of doctors could complete the filling of 33 items of information within 2 min, which is a reduction in time compared to the past. The timely reminders from the system reduced the rate of missed cases by doctors; the analysis using interrupted time series method showed an average reduction of 4.4037% in the missed-case rate. This study proposed a knowledge-based infectious disease decision support system based on real medical records and knowledge rules, and its effectiveness was verified. The system improved the management of infectious diseases, increased the reliability of decision-making, and reduced the rate of underreporting.

## Introduction

Infectious diseases have always been a critical public health challenge for human society. The COVID-19 pandemic posed a substantial threat to the life and health of all mankind and caused considerable economic losses to countries around the world^1,2^. As the first line of defense in the diagnosis and treatment of infectious diseases, hospitals' real-time monitoring and early warning capabilities for infectious diseases have a major impact on the success and efficiency of the entire infectious disease prevention and control work. Early infectious disease warning and reminders can affect the treatment effect and prevention and control of infectious diseases considerably^3,4^. Studies^5,6^ have mainly relied on laboratory test results for monitoring and early warning. For example, during the COVID-19 pandemic, medical professionals and hospital managers were alerted by critical values when nucleic acid test results were positive^7^. Previous studies^8,9^ have shown that electronic reminders based on test results have important implications for disease diagnosis and treatment, especially in the field of infectious diseases, as they enable early isolation and management of infectious diseases. However, relying solely on test results for accurate and comprehensive infectious disease early warning is inadequate, as infectious disease diagnosis requires information from multiple sources, such as symptoms, signs, diagnosis, and medical history throughout the patient's entire medical record. With the gradual proliferation of Electronic Health Record Systems (EHRS), EHRS has become a management system for daily applications in hospital outpatient and inpatient departments. Several studies have shown that EHRS can accelerate clinical information flow, promote medical data integration, and improve the efficiency and quality of medical services^10,11^. However, current EHRS are mainly designed for clinical practice rather than public health purposes^12^. Therefore, it is difficult to directly monitor infectious diseases according to relevant diagnostic criteria using EHRS, and manual retrieval and report generation are required, which is time-consuming and prone to omissions.

After the outbreak of SARS in 2003, in addition to monitoring infectious diseases, China established a direct reporting system for infectious diseases. China divides infectious diseases into three categories, A, B, and C, based on their impact and harmfulness, with A being the highest priority. When a case is diagnosed as a certain infectious disease, it is required to be reported to the Chinese Infectious Diseases Reporting System (CIDRS) within a designated time frame. Clinical doctors are required to report to the hospital's disease control management department through an electronic medical record system, after which the hospital's disease control department reviews the reported information before transcribing it into CIDRS^13^. Unfortunately, given the lack of uniformity among the electronic medical record systems of various hospitals, it is difficult to connect them all to the national system, and transcription reports involve time-consuming and error-prone manual operations. At present, for infectious disease reporting based on electronic medical records, some medical institutions in China choose to judge whether a disease is an infectious disease by monitoring diagnosis names, and monitor cases that are diagnosed as infectious diseases but are not actually reported to a nationwide authority.

In order to make electronic medical record systems more intelligent, preliminary research^14^ extended Clinical Decision Support Systems (CDSSs) on the basis of electronic medical records. A CDSS is a system that provides intelligent processing of disease-specific and patient-specific data for clinical physicians, patients, and other individuals^15^. Several diverse types of CDSSs can be found in literature^16,17^, such as diagnostic tools, expert systems, and workflow support. CDSSs have provided considerable assistance in clinical practice, with over 62% of trials demonstrating enhancements in practitioner performance, reminder systems, drug dosage systems, and disease management systems. At present, more than one-third of CDSS applications are mainly used in chronic disease management, treatment prediction, and risk assessment^17^. For instance, Dalal et al.^18^ and Khan et al.^19^ developed treatment risk alerts for elderly patients, considering factors such as suitability for catheter insertion, likelihood of physical function recovery, and potential drug interactions. In Tao et al.'s study^20^, CDSS was shown to reduce the average length of hospital stays by almost one day, demonstrating their potential to effectively enhance medical efficiency and improve clinical practice. In contrast, some studies have analyzed how CDSS introduction interferes with physician workflows, with ineffective alarms and frequent alerts leading to physician fatigue and oversight^21^.

With respect to the alerting methods used in CDSS applications, Park al.'s research^22^ used interruptive alerts in drug interaction warnings, which directly interrupted the clinical physician's workflow with pop-up notifications. Binh et al.^23^ used list-style alerts to remind clinical physicians to complete preventive care orders. The alerting methods in previous research were all single-mode reminders, lacking the ability to set varying levels of alerts. As for the interpretability of CDSS, in our preliminary research^24^, we employed the Multiple Infectious Disease Diagnostic Model (MIDDM) to provide warning alerts based on medical records. However, owing to the limitations of machine learning in providing evidence-based explanations, clinical physicians lack reliable bases for alerts, which can have noticeable impacts on patients, especially in cases of misdiagnosing infectious diseases.

As of now, CDSS applications with better interpretability are based on rule-based knowledge bases. However, previously, CDSS rule libraries were established based on clinical experts, which were then hardcoded by technical developers into the system's programming backend, making them difficult to modify. Thus, CDSS rules are challenging to develop, update, and maintain^25^. Consequently, one of the concerns to be addressed in this research is how to extract rules based on clinical knowledge as well as how to supplement, update, and maintain these rules through the system.

Therefore, in order to address the problems of passive and single-diagnosis or single-test infectious disease monitoring, and the complete dependence on manual active reporting of infectious diseases, this study establishes an infectious disease monitoring and decision support system to assist clinical physicians in infectious disease decision-making. Based on clinical guidelines and real medical record knowledge maps, complex rules are generated to simultaneously monitor patient medical records, examination reports, and other results, and comprehensively judge whether they meet the national diagnostic criteria for infectious diseases. This meets the needs of infectious disease monitoring and alerts doctors to cases that meet the positive indicators of infectious diseases, assisting them in making diagnostic decisions. In addition to physician alerts, a management backend is also established for management departments to manage and monitor infectious disease reporting.

## Methods

### System design and implementation

This study focused on a large comprehensive hospital and its three branch hospitals in China, having a total of 2353 beds and a daily average outpatient and inpatient volume of 17,000. Based on the hospital's electronic medical record system and laboratory information system, an Infectious Disease Monitoring and Decision Support System was established. The system monitors all patient medical record information during their visit, including age, gender, vital signs, medical history, laboratory test results, diagnosis, and radiographic examination results. As the hospital system generates a random ID for each patient and saves it in a secure server area, the anonymity of all patients was maintained. Data analysis was conducted using Excel to calculate the proportion of time intervals. The R 4.2.2 software was used to analyze the changes in slope before and after the system's implementation as well as the immediate level changes at the intervention points. The computers used by hospital doctors had the same configuration, i.e., Intel(R) Core(TM) i7-7500U CPU @ 2.70 GHz, 8 GB of running memory, and a total hard disk size of 256 GB.

The study was approved by the Medical Science Research Ethics Committee of Peking University Third Hospital (serial number: IRB00006761-M2022287). The Peking University Third Hospital Medical Science Research Ethics Committee approved our study and waived the need for informed consent. We confirm that all the experiment protocols for including humans were in accordance with the guidelines of national/international/institutional requirements or the Declaration of Helsinki.

### System architecture and workflow design

**Figure 1:**
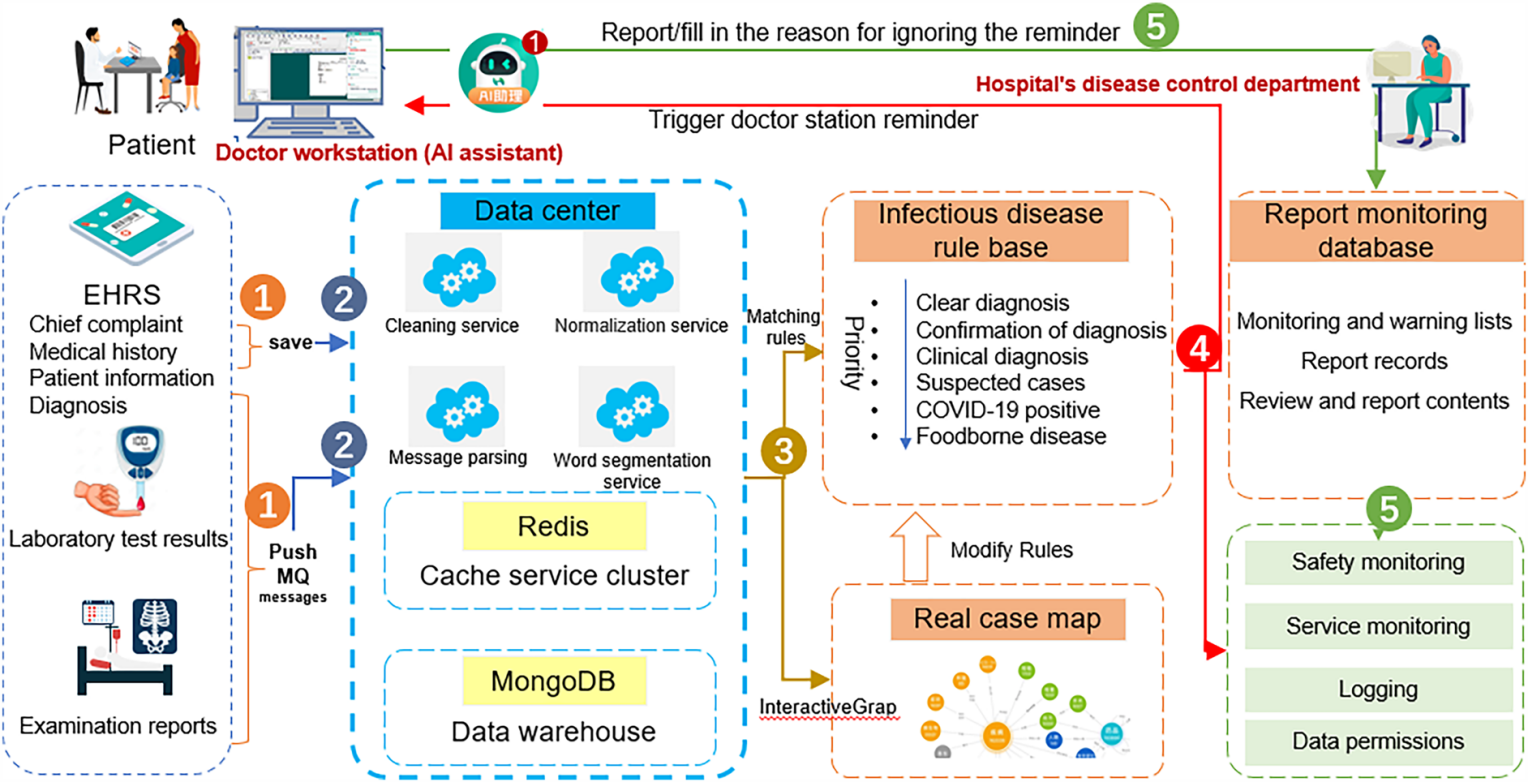
Data flow of the infectious disease monitoring and decision support system.

The hospital selected for this study has a good level of informatization and has aggregated data from multiple systems, such as EMR, medical orders, laboratory tests, and inspections, to the data center. All unstructured data are processed into structured data in the data center. The Infectious Disease Monitoring and Decision Support System can directly connect to the data center to obtain the full range of data, including medical records and laboratory tests. The system consists of two parts: the decision support end (AI assistant) for doctors and the monitoring and management end for the hospital's disease control department. The doctor's decision support end (AI assistant) is integrated with the outpatient and inpatient doctor station, and the AI assistant system is automatically opened when the doctor logs in to the doctor station, with the listening status indicated by a small AI Assistant icon. The monitoring and management end of the hospital's disease control department is a web-based page that can only be accessed by authorized personnel and is dedicated for management department use. The system data flow is shown in Fig. 1.

#### Establishment of a complex rule library based on knowledge map and diagnostic criteria

**Figure 2:**
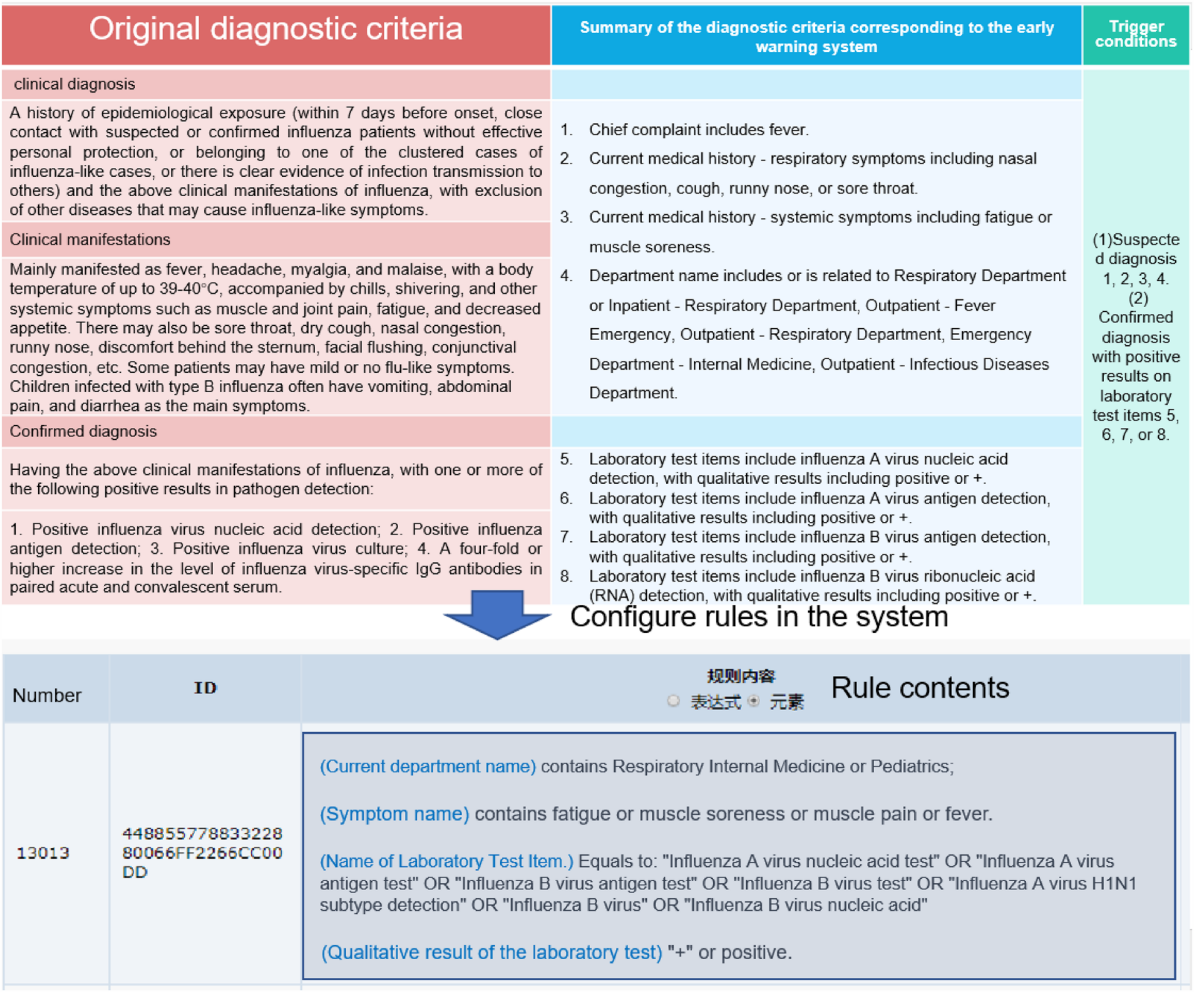
Records of rules extracted for influenza and visualization of rule configuration in the system.

Infectious disease diagnoses can be classified into suspected cases, clinically diagnosed cases, confirmed cases, and carriers of pathogens^26,27^. First, based on diagnostic criteria, two groups of senior experts from infectious disease departments such as respiratory, pediatrics, and dermatology summarized the guidelines and then cross-verified and discussed to form standardized content corresponding to the medical record data source. For example, the rule extraction for influenza is shown in Fig. 2. In Fig. 2, the extracted rule criteria are based on "Standards for Diagnosis of Statutory and Other Key Monitored Infectious Diseases in the Health Industry of the People's Republic of China". The expert group conducting cross-validation found it challenging to list all the symptoms or physical examination descriptions of doctors in one go during symptom supplementation. This led to several omissions in the monitoring process. Therefore, considering the actual operation of the hospital, this study uses a graph generated based on real historical data to analyze the doctor's writing habits and actual medical record data for rule revision and supplementation, so that the monitoring rules can be triggered in real clinical work.

**Figure 3:**
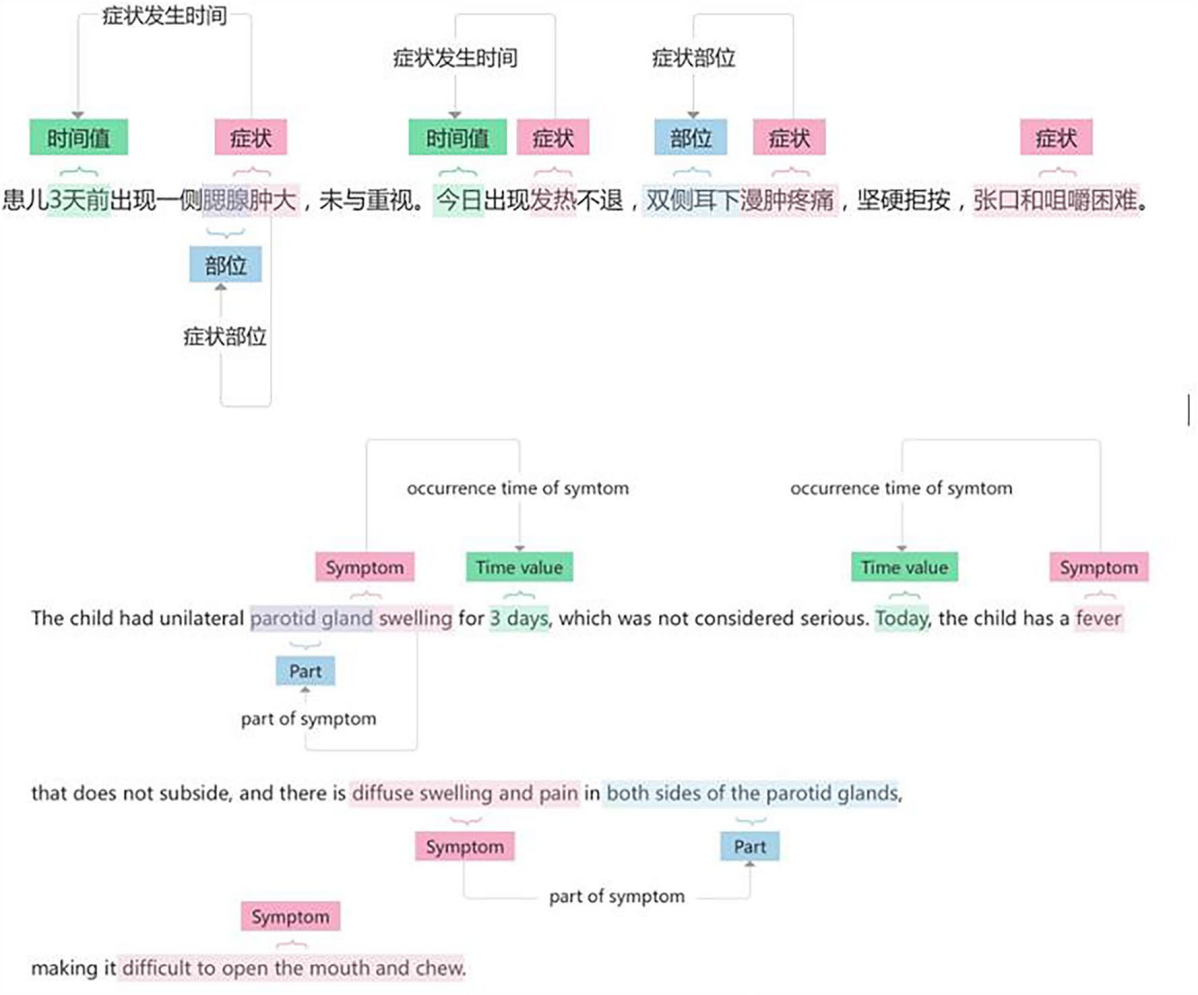
Natural language processing and structured processing of infectious disease medical records (in Chinese and English).

In this study, we employed natural language processing techniques^28^ established in previous research as the foundation. For the non-structured portion of real-world clinical data, we utilized the BiLSTM-CRF network^29^, an open-source method with effective sequence labeling performance. We combined this with rule-based models and other methods to extract information from the original electronic medical records. In this process, first, tokenized and serialized text were input into the BiLSTM layer, combining the results of forward and backward hidden states to generate the BiLSTM output^30^. Subsequently, the output of BiLSTM was fed into the CRF layer, forming the BiLSTM-CRF network structure. This architecture takes advantage of both BiLSTM and CRF: the BiLSTM, as a bidirectional LSTM component, effectively preserves the contextual information of the entire sentence, enabling feature extraction from the sentence, while the CRF layer leverages the ability to learn constraint information from the training data, further enhancing the accuracy of information extraction, as illustrated in Fig. 3.

**Figure 4:**
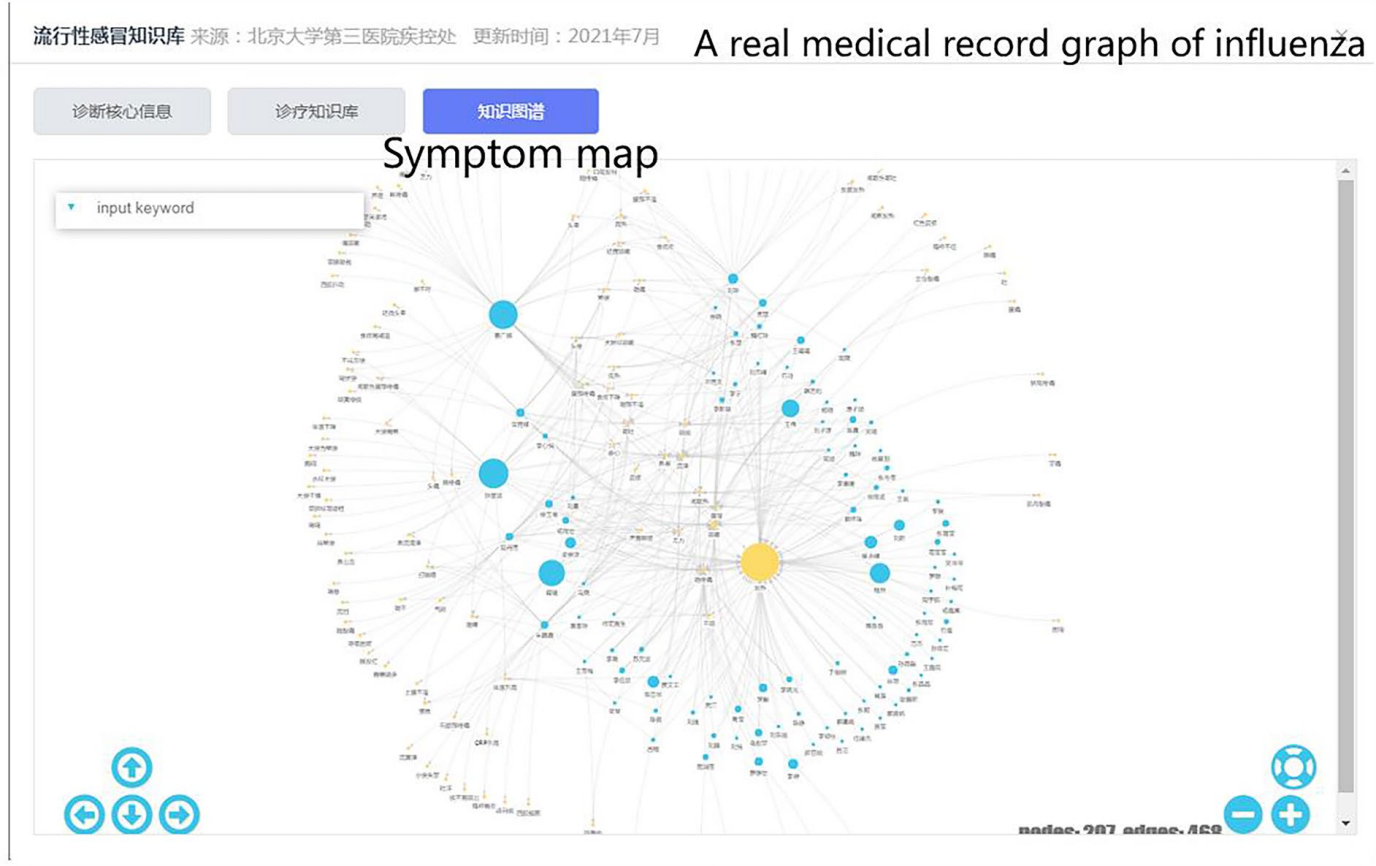
Based on the actual historical medical records of the hospital, a real medical record graph of influenza is generated.

Based on the structured data, a knowledge map was drawn using the open-source InteractiveGraph graph data interactive visualization analysis framework^31^, as shown in Fig. 4. Separate graphs are drawn for outpatient and inpatient cases, displaying the symptoms and doctors’ notes that appear in the real medical records pertaining to an infectious disease. For high-frequency symptoms, larger graphics are used, and the occurrence frequency is indicated on the connecting line. Considering the readability of the graph and the rotation of doctors, the data is displayed for the past six months, making it convenient for rule review experts to supplement rules at any time.

#### Decision support for various types of infectious diseases

**Figure 5:**
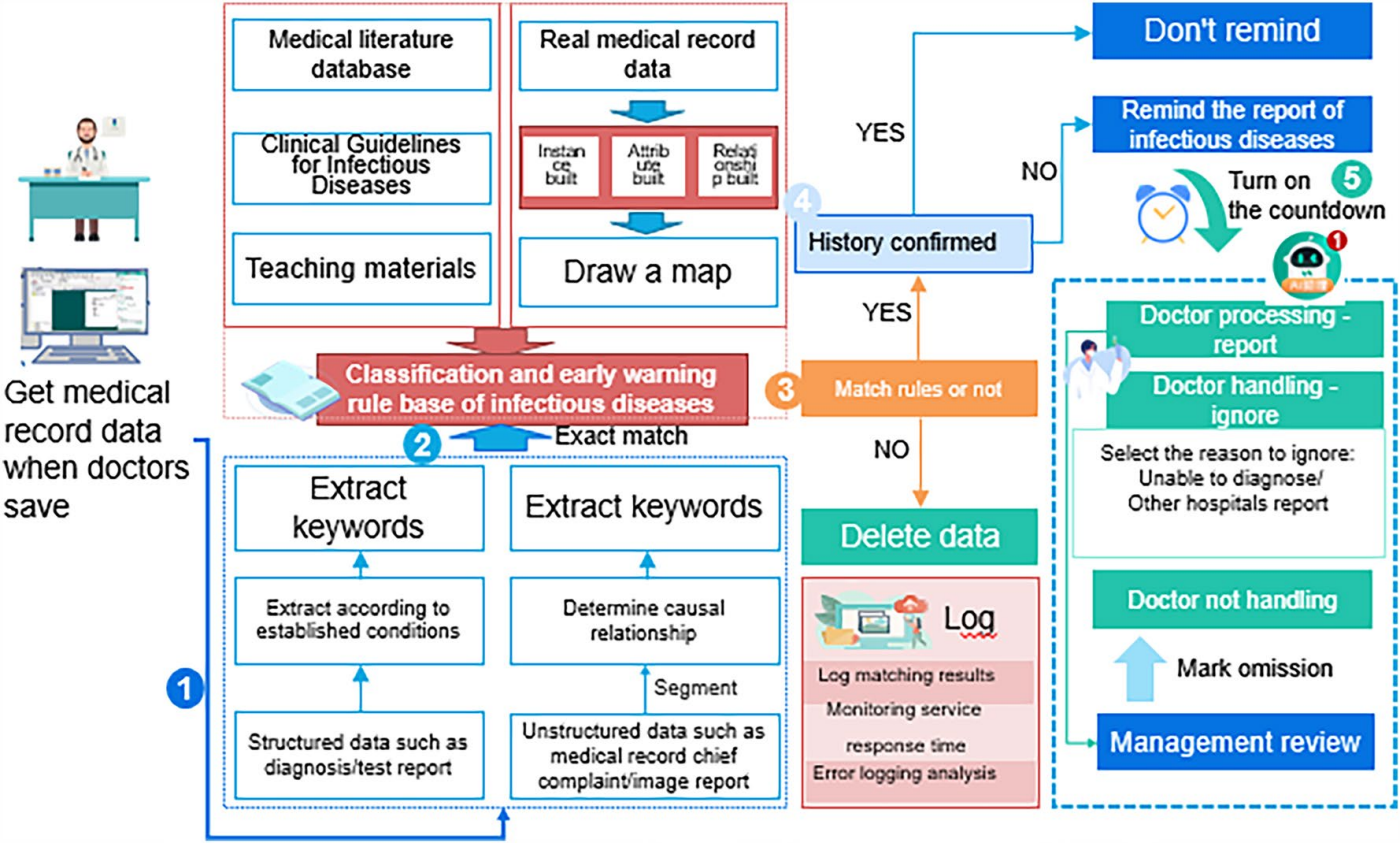
System rule matching process.

**Figure 6:**
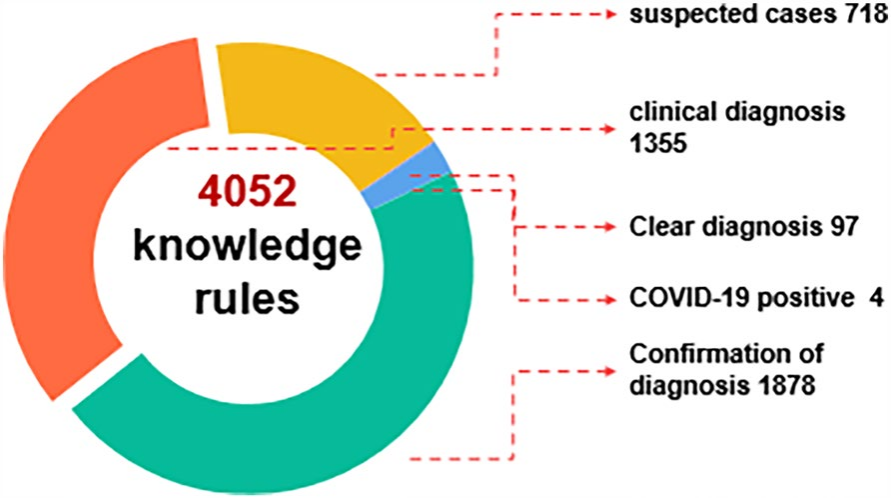
Infectious disease alert rules are counted by type.

The system provides infectious disease warning reminders of various severity levels based on the rule library. The AI assistant on the doctor's decision-making end is integrated with the doctor's workstation. When a doctor saves a medical record, the text structuring service is called immediately to perform word segmentation processing. It then matches a variety of level rules in the knowledge rule library and makes a diagnosis directly (the doctor enters the diagnosis name directly in the medical record diagnosis). The highest priority is given to a direct diagnosis, followed by confirmed diagnosis, clinical diagnosis, and suspected cases. The rule matching and decision support process is shown in Fig. 5, and the number of rules is shown in Fig. 6. The number of rules varies considerably for different diseases owing to the influence of their clinical diagnostic guidelines and disease complexity. Diseases such as epidemic encephalitis B, measles, and epidemic hemorrhagic fever are associated with a higher number of rules, while diseases like seasonal flu and hand, foot, and mouth disease have fewer rules, each with 27 rules.

Among the diseases, epidemic encephalitis B has the highest number of rules. Specifically, the diagnostic criteria for epidemic encephalitis B are divided into mild, moderate, severe, and extremely severe cases. The rules also involve a combination of physical examination findings and chief complaints or medical history, leading to a considerable increase in the number of rules.

Furthermore, during the COVID-19 pandemic, a new classification was added for monitoring positive COVID-19 test results. This classification has the lowest priority. When new evidence is made available to the clinical diagnosis or confirmed diagnosis criteria, the positive test result will be updated directly under the confirmed diagnosis classification. For example, when the COVID-19 antigen test is positive, it triggers the positive monitoring. When the nucleic acid test is positive and the patient has flu-like symptoms, it satisfies the confirmed diagnosis rule, triggering a reminder under the confirmed diagnosis classification, which covers the original positive monitoring.

Clear diagnosis decision support: Doctors issue a clear diagnosis in the doctor's workstation, and the AI assistant automatically retrieves the diagnostic message. After matching the diagnostic ICD-10 code with the diagnosis set by the infectious disease monitoring rules, the AI assistant will remind the doctor to report the diagnosis once the matching is successful.

Confirmation of diagnosis or clinical diagnosis decision support: Matching the patient's complaints, medical history and laboratory results, the system provides reminders. For example, for confirmed diagnosis of influenza, when the patient's test result is positive for influenza virus antigen or "+", the AI assistant will actively remind the doctor to confirm the patient's diagnosis of influenza and to diagnose and report the medical record in a timely manner.

Decision support for suspected cases: Suspected cases mainly include those who meet the requirements of the guidelines, such as those with medical history, contact history, or obvious symptoms. The system triggers the rule for suspected cases accordingly. For example, for suspected cases of epidemic parotitis, when the doctor writes "bilateral parotid swelling for 3 days" in the medical record, the system calls the structured service to match the segmented result with the rule library. The AI assistant will actively remind the doctor that the patient is a suspected case of epidemic parotitis and suggest other indicators of the disease. The AI assistant also provides a knowledge base for clinical doctors in non-infectious disease departments to understand infectious diseases.

**Figure 7:**
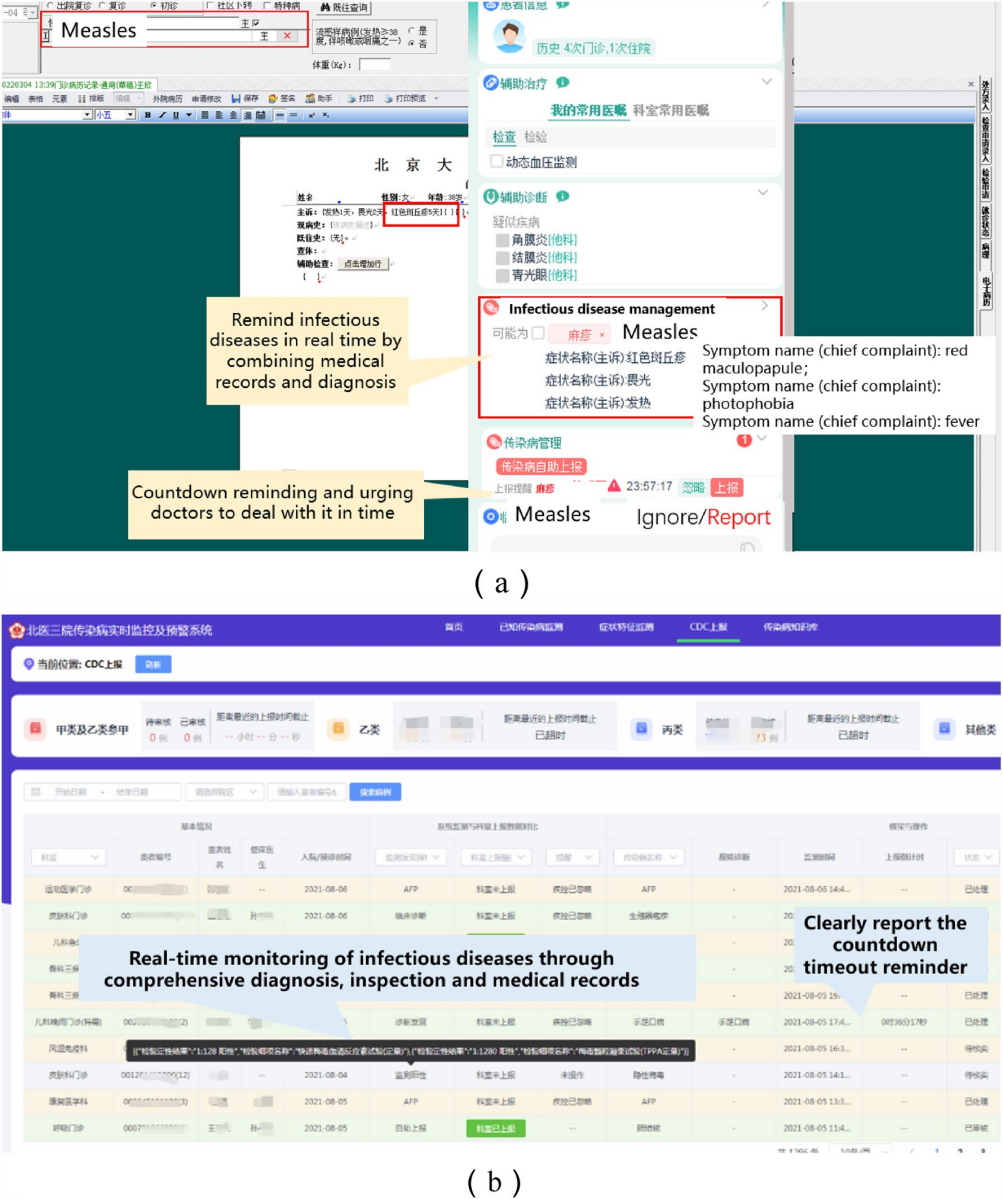
Integration of the infectious disease surveillance and decision support system with the doctor workstation provides real-time reminders during medical record writing (**a**). Public health personnel can review each alert record and modify the content of the report through the system management interface (**b**).

After the doctor sees the reminder and confirms the diagnosis for the infectious disease medical record that needs to be reported, the system automatically fills in the report content to reduce the doctor's input workload. The system also automatically checks the data integrity. After the doctor confirms and submits the report, it is sent to the hospital's disease control department. The infectious disease control personnel monitor the reporting, audit process, and status in real time. For situations where a diagnosis cannot be made and reporting is not required, the system provides an ignore option for interaction between doctors and infectious disease control management personnel. The ignore function avoids missed reporting and duplicate reporting. The reasons for ignoring an infectious disease diagnosis include old lesions and carriers of pathogens that do not need to be reported. This solution effectively solves the problems of missed reporting and misreporting in active reporting methods. The screenshots of the doctor-side and management-side systems are shown in Fig. 7.

#### Interruption-based and non-interruption-based reminder decision-making

**Figure 8:**
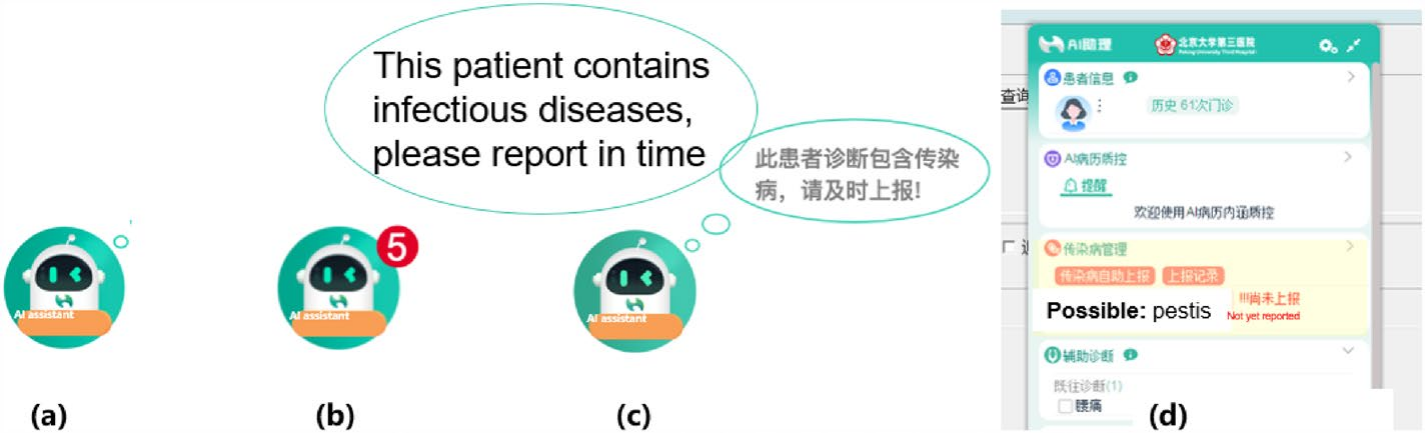
(**a**) No reminder state is represented by the AI assistant icon. (**b**) When triggered by a rule, the AI assistant icon displays a numbered reminder in the upper-right corner. (**c**) The bubble represents a non-interruption-based reminder. (**d**) The pop-up window represents an interruption-based reminder from the AI assistant, which will be highlighted and flash.

According to China's classification and management requirements for infectious diseases, medical institutions are required to report Class A infectious diseases to CIDRS within 2 h, and Class B and C infectious diseases within 24 h^32^. Therefore, the reminder system is designed based on the classification and management requirements of infectious diseases. To avoid affecting the doctor's diagnosis of current patients, for rules that require inspection results to trigger, the system triggers infectious disease warning prompts for patients that the doctor has seen when they return to the patient list after ending the current patient's consultation. When a reminder needs to be sent to other doctors for follow-up consultation or reporting, the system automatically combines the reporting and reminder results to avoid duplication. A variety of reminder methods are shown in Fig. 8.

Popup interrupt reminder: For Class A infectious diseases that have already been diagnosed, the decision support system interrupts the doctor's work directly in the form of a popup window, and the small icon of the decision support system expands to show details, highlighting and flashing the reason for the interruption, such as "Monitoring reason: diagnosis includes Pestis, disposition reminder: report required within 2 h", to draw enough attention from the doctor.

Non-interruptive bubble reminder: For Class B and C infectious diseases, after a clear diagnosis has been made, the AI assistant icon of the decision support system appears in a bubble dialogue box, reminding the doctor to report the infectious disease and automatically starting a 24-h countdown. The doctor can click on the AI assistant to expand the report card for reporting. For infectious diseases that meet the clinical diagnosis and suspected conditions mentioned in "Decision support for various types of infectious diseases" but do not have a clear diagnosis, the bubble dialogue reminder is still triggered. After the doctor clicks on the AI assistant, they can view the specific rule content that is met, such as "Monitoring reason: chief complaint includes bilateral parotid gland enlargement, disposition reminder: report or ignore". The doctor can see this reminder until it is resolved. After the doctor reports or fills in the reason for ignoring, the disease control manager can view the interaction content in the monitoring system.

No reminder: For patients who have reported infectious diseases within the last 30 days, no reminder is given during their follow-up consultations.

#### Management department monitoring and inspection

For the hospital's disease control department, the system mainly meets the requirements of in-hospital infectious disease monitoring, content review, and abnormal symptom monitoring and analysis. For content review, disease control department staff can revise the content and generate a revision mode. Doctors can view the reasons for the modification, which improves the quality of reported content. For infectious disease monitoring, a list of detailed reasons for triggering rules at all doctor stations in the hospital will be displayed. Disease control department staff can mark and make notes on cases of missed reporting where doctors have not processed the reminders and alerts.

### Reducing the intervention of invalid alarms

Setting trigger restrictions for specific diseases and specialties: For sexually transmitted diseases such as chlamydia trachomatis infection and gonorrhea, diagnosis and reporting must be done by specialists with diagnostic privileges in dermatology, obstetrics and gynecology, and urology, as per the diagnostic guidelines. For hand, foot, and mouth disease, diagnosis must be performed by pediatricians. Therefore, for other departments, the system will block the trigger rule reminders. For example, even if an ophthalmologist issues such a diagnosis, it cannot be reported as an infectious disease diagnosis, and the reporting button will be automatically blocked to reduce the misreporting rate for non-specialists.

Setting the non-monitoring time range for follow-up visits: For patients who have follow-up visits within a certain period, the system can be set to exclude monitoring. For example, for patients with viral hepatitis who need regular medical visits and follow-up visits to obtain medication, the system can be set not to monitor the diagnosis name "viral hepatitis," and the time period can be set as permanent, 3 months after reporting, or 6 months after reporting. The time period can be customized.

Monthly rule maintenance and upgrades: For diseases with extremely high trigger frequency but relatively low actual diagnosis rate, the system will disable the rule. For example, the suspected rule for bacterial dysentery is that the chief complaint includes diarrhea. However, there are many diseases that present with diarrhea, which can interfere with the diagnosis of bacterial dysentery, so the suspected rule will be disabled. The upgraded rule will require clinical diagnosis and confirmation through fecal smear examination, which shows a large number of red blood cells, a small number of white blood cells, Sha-Ko-Liden crystals, or visible amoeba trophozoites and/or cysts in the tissue.

### Evaluation of system effects

The system effects will be evaluated from three perspectives. First, the effectiveness of various types of decision support reminders, including the overall number of triggers, effectiveness rate, and analysis of invalid situations need to be validated. Second, the usability of the system will be analyzed, including the distribution of time from patient arrival to diagnosis, the distribution of time for timely reporting after system reminders, and the time taken by doctors to fill out the report card. Third, interrupted time series methods^33^ will be used to compare the proactive reporting mode of doctors before the system is online with the decrease in missed reports after the system is online.

### Ethics approval and consent to participate

The study was approved by the Medical Science Research Ethics Committee of Peking University Third Hospital (serial number: IRB00006761-M2022287). Informed consent from the patients was exempt due to the retrospective nature of the study. The ethics committee (Peking University Third Hospital Medical Science Research Ethics Committee) approved the study abandoned informed consent. We confirm all the experiment protocol for involving humans was in accordance to guidelines of national/international/institutional or Declaration of Helsinki in the manuscript.

## Results

### Rule trigger and false alarm situation

The system was launched in December 2021 and was fully implemented in a large comprehensive hospital and its three branch campuses in China, monitoring and providing alerts for all outpatient, emergency, and inpatient cases. The system is utilized by 1146 doctors across 30 clinical departments in the hospital. These include common infectious disease departments such as Pediatrics and Respiratory departments as well as screening departments where infectious diseases may be identified. For the retrospective research analysis, we chose a stable period with minimal impact from the COVID-19 pandemic, i.e., December 2021 to November 2022, covering a total of 12 months. December 2022 was excluded from the study because of major changes in China's epidemic prevention policies^34^, which affected the volume of fever-related visits.

During the retrospective research period, the system analyzed 4,497,091 medical records and triggered 12,027 monitoring and warning messages. Among these warnings, 54.63% (n = 6570) were related to infectious diseases and reported, 43.80% (n = 5269) were considered valid by doctors but not reported, and 1.56% (n = 188) were deemed invalid by doctors and not reported. Within the reported warnings, 87.32% were related to COVID-19 with positive results in IGG, IGM antibodies, and nucleic acid testing. The monthly breakdown values of warning results are listed in Table 1, categorized based on the reminder outcomes.

**Table 1:** Analysis of infectious disease alerts by the infectious disease alert system from December 2021 to November 2022.

Time period	Total	COVID-19 monitoring positive IGG/IGM/PCR	Suspected	Suspected reported	Clinical diagnosis	Clinical diagnosis reported	Confirmed diagnosis	Confirmed diagnosis reported	Direct diagnosis discovery	Direct diagnosis discovery reported
December 2021	1463	547	1	0	6	0	59	32	850	814
January 2022	2486	160	0	0	2	0	29	18	2295	2261
February 2022	1021	157	0	0	6	0	38	18	820	787
March 2022	1228	353	6	0	17	1	40	17	812	772
April 2022	804	460	14	0	13	0	56	14	261	218
May 2022	613	440	8	0	12	0	26	12	127	102
June 2022	544	378	7	0	11	1	46	27	102	68
July 2022	689	438	16	0	9	0	35	18	191	154
August 2022	1083	553	6	0	7	1	32	22	485	437
September 2022	855	442	9	0	10	0	42	27	352	316
October 2022	645	366	9	0	10	0	33	15	227	201
November 2022	596	307	6	0	6	0	63	35	214	182

During the retrospective study, among the 12,027 monitoring alert messages triggered, the gender ratio was 1.108:1, with an almost equal proportion of males and females. The disease category and reporting situation for the month of February 2022, which had the highest monitoring volume, are listed in Table 2. Combining Tables 1 and 2, it was found that the suspected and clinically diagnosed rules had reporting rates of 0% and 2.75%, respectively, resulting in a considerable number of ineffective alerts, and doctors marked them as "not meeting the diagnostic criteria". The suspected rule mainly concerned mumps, with the rule being [{"Symptom Name (Present Illness or Chief Complaint)": "Swollen Parotid Gland"}]. The clinical diagnosis rule was mostly based on medical history or symptom monitoring, and based on the actual alert situation, the diseases for which alerts were raised most frequently were genital herpes, condyloma acuminatum, and hand, foot, and mouth disease. The rule for genital herpes was [{"Symptom Name (Present Illness)": "Skin Blister"} and {"Body Part Name": "Vagina"}], most of which were actually diagnosed as shingles, so it is necessary to optimize the rules jointly with experienced clinicians, further subdivide genital herpes, or choose not to use this rule to trigger alerts.

**Table 2:** Alerts and reports by disease category in February 2022.

Disease category	Total alerted cases	Reported cases	Disease category	Total alerted cases	Reported cases
Viral hepatitis	8	5	Brucellosis	1	0
Tuberculosis	51	13	Mumps	3	3
Influenza	2247	2234	SARS	2	0
Hand, foot, and mouth disease	3	3	Gonorrhea	7	5
Syphilis	10	2	Leprosy	1	1
Scarlet fever	1	1	Chickenpox	16	15
Schistosomiasis	2	1	Chlamydia trachomatis infection of reproductive tract	26	21
COVID-19	102	0	condyloma acuminatum	1	0
Genital herpes	2	0	Bacterial dysentery	3	1

Of the confirmed diagnoses, 51.10% of the alerts were effectively reported in a timely manner, while the remaining 48.90% were marked as reminders being effective but not requiring reporting in the system, as the patients had already been diagnosed in other hospitals or were just returning for follow-up visits. For the diagnoses directly made by doctors in the medical records as presented in Table 1, the effective reporting rate of doctors was 93.70%, and the remaining unreported cases were mainly related to follow-up prescriptions. Overall, this convincingly demonstrates that the alert system has a high effective rate.

### System effectiveness analysis

A total of 2523 confirmed infectious disease cases that were randomly sampled and completed reporting from December 2021 to May 2022 were analyzed with regard to the timeliness of diagnosis, effectiveness of reminders, and efficiency of card filling.

**Figure 9:**
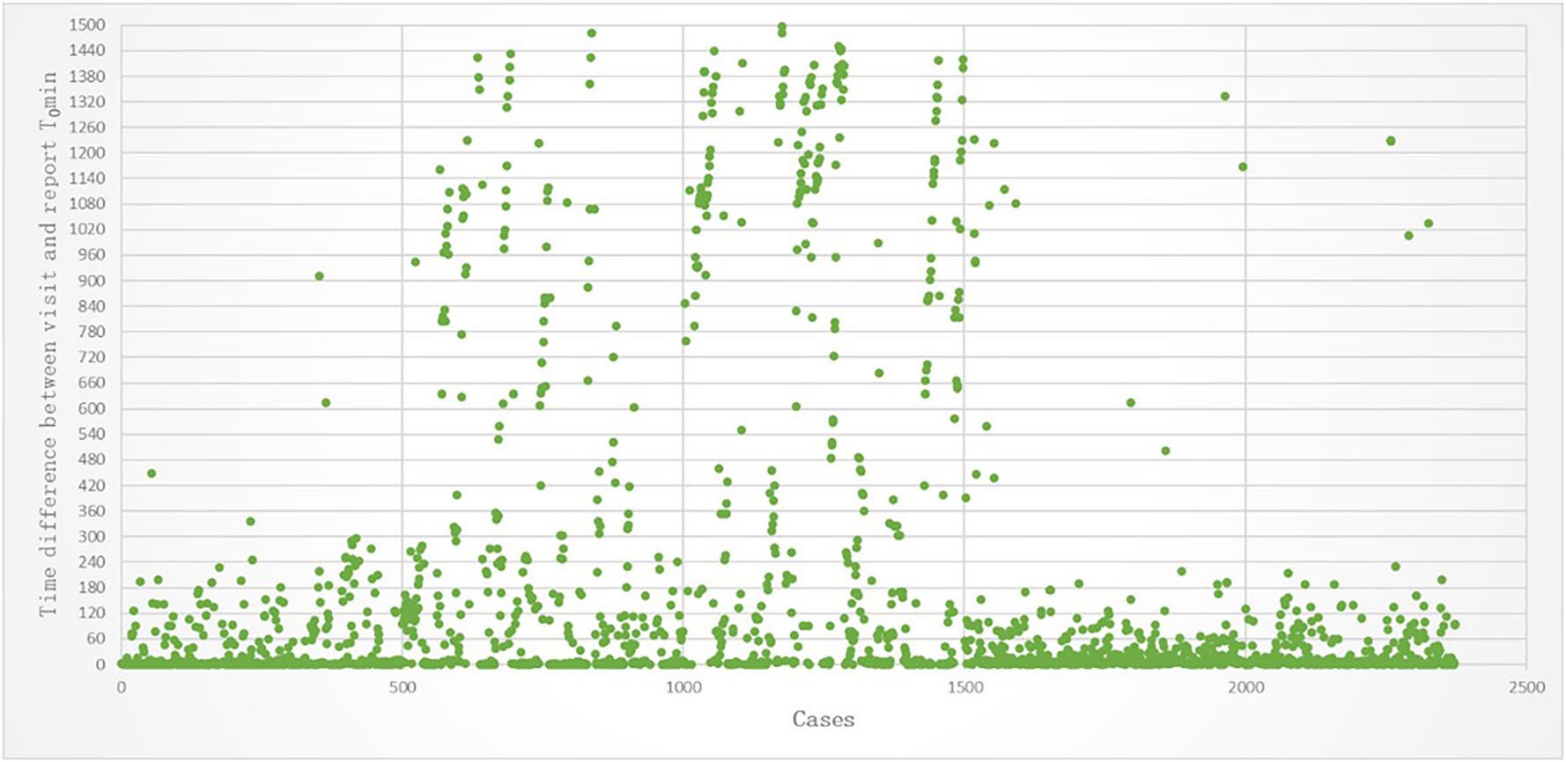
Distribution of time interval between onset and diagnosis for influenza patients.

Timely diagnosis of infectious diseases will substantially reduce the risk of nosocomial infections caused by patients waiting for diagnosis. The time at which patients were first seen by doctors at the doctor station was taken as the visit time (${T}_{visit}$), and the time at which the system triggered an alert was taken as the monitoring time (${T}_{monitoring}$). Equation (1) was used to calculate ${T}_{0}$, which represents the interval between the patient's visit and the system's alert for infectious diseases, indicating the timeliness of diagnosis. The overall analysis is presented in Table 3. For reproductive tract chlamydia trachomatis infection, gonorrhea, and syphilis, the first alert time was more than 24 h, mainly because of the rule that positive indicators in the test results were included, and the test report takes 3–4 days to issue. For some patients with pulmonary tuberculosis, hospitalization is required because of lung shadows and common differential diagnosis of lung cancer, and more tests or pathological results are needed for confirmation^35,36^. In addition, pulmonary tuberculosis is sometimes discovered through physical examination, which takes a long time to report. Based on the symptoms of respiratory infectious diseases in the early stages of the SARS epidemic in 2003 and the COVID-19 pandemic in 2019^37,38^, respiratory infectious diseases are the main cause of nosocomial transmission risk for hospitals. Therefore, the shorter the diagnosis time of respiratory tract diseases, the higher the reduction of nosocomial transmission risk. As shown in Fig. 9, 60% of influenza cases can remind doctors that patients meet the diagnostic criteria for influenza within 30 min. 4.5% of influenza cases have a diagnosis time of more than 24 h, mainly owing to hospitalized patients who were initially diagnosed with a disease other than influenza, then later tested for influenza antigen during their hospital stay, and determined to have influenza. For genital chlamydia infection and gonorrhea, the average time of ${T}_{0}$ exceeds 24 h, mainly as it requires the support of test results, which take 3 days to obtain. For tuberculosis, 50% of tuberculosis patients are detected through medical examinations, wherein it takes longer to generate reports than regular outpatient and inpatient visits.

**Table 3:** Time interval between patients' visit to the system for monitoring and warning infectious diseases ${T}_{0}$.

	Influenza	Hand, foot, and mouth disease	Epidemic parotitis	Genital chlamydia infection	Gonorrhea	Infectious diarrhea	Tuberculosis	Varicella	Viral hepatitis	Bacterial dysentery	Scarlet fever
Total cases	2375	6	1	41	8	4	11	40	2	5	1
${T}_{0}\le 30$ min	1429	4	0	0	0	0	0	24	0	0	0
${30<T}_{0}\le 60$ min	137	0	0	0	0	4	0	3	0	5	0
${60<T}_{0}\le 90$ min	118	0	0	0	0		0	2	0	0	1
${90<T}_{0}\le 120$ min	85	1	1	0	0	0	0	0	0	0	0
${2<T}_{0}\le 24 \mathrm{h}$	498	1	0	0	0	0	0	11	2	0	0
${24 \mathrm{h}<T}_{0}$	108	0	0	41	8	0	11	0	0	0	0
Average time to diagnosis	74% of influenza cases can be diagnosed within 2 h	57 min	1 h 44 min	4 days 6 h	3 days 23 h	50 min	5 days 4 h	62 min	9 h	45 min	77 min

1 $${T}_{monitoring}-{T}_{visit}={T}_{0}.$$

**Figure 10:**
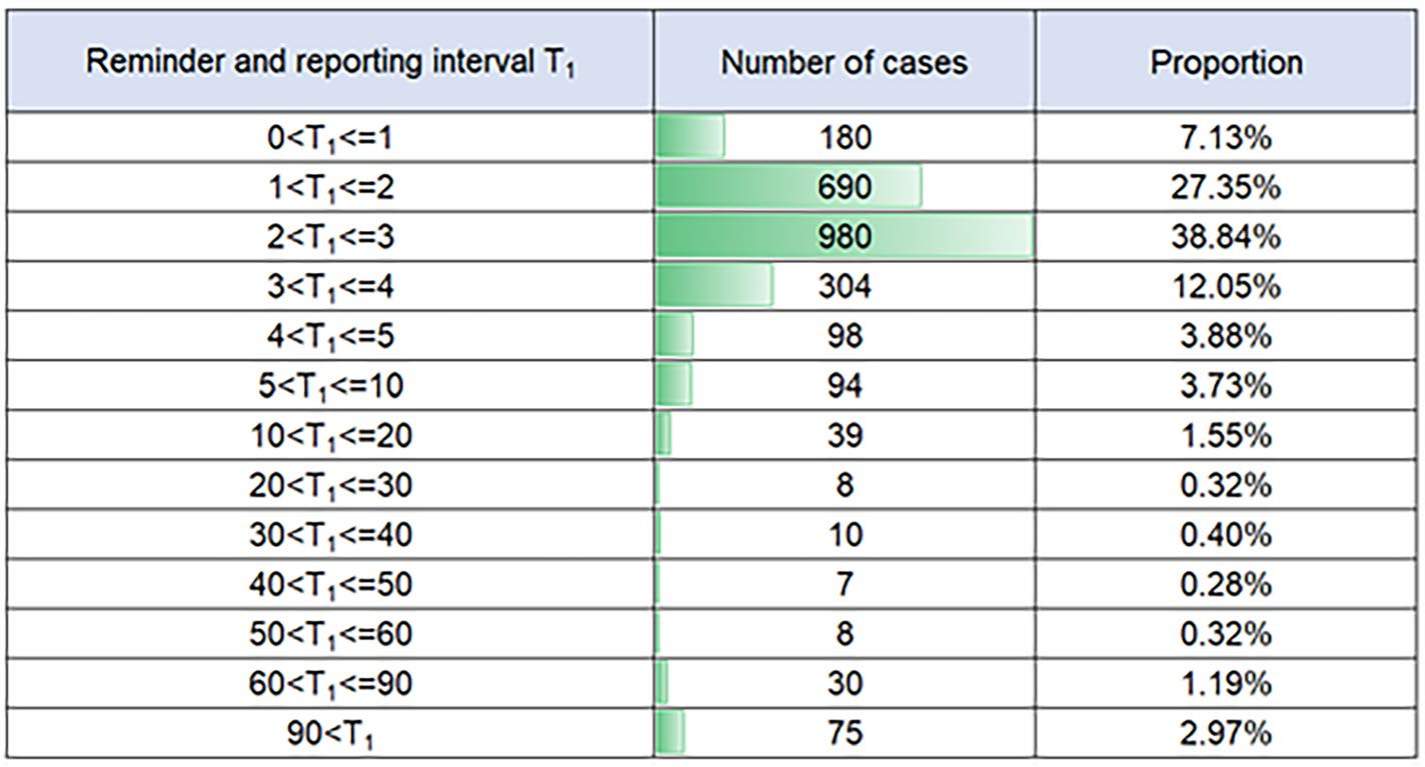
Analysis of time interval between reminder and reporting by doctors.

Timeliness analysis of reminders is important. The time interval between receiving a reminder and reporting it by doctors is analyzed. The triggering time of the alert in the system is taken as the monitoring time ($${T}_{monitoring})$$, and the time when the doctor clicks the "report" button is taken as the reporting time ($${T}_{report})$$. Equation (2) is used to calculate $${T}_{1}$$ to obtain the time difference between the reminder and the report, which indicates the timeliness of the reminder. The results are shown in Fig. 10. 89.26% of infectious disease cases can be confirmed and reported within 5 min after the reminder is received. According to the system's reminder setting, doctors are reminded of positive results of previously treated patients after they complete the current patient's consultation, so some cases may take more than 5 min to report. For 2.97% of cases that took longer than 90 min, it was mainly due to positive test results being reported during doctor shift changes or holidays, and other doctors had to handle them.

2 $${T}_{report}-{T}_{monitoring}={T}_{1}$$

**Figure 11:**
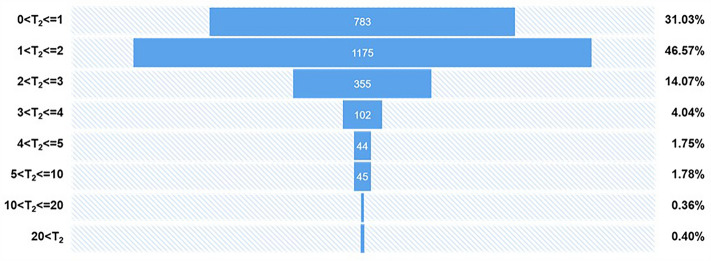
Time for doctors to fill out the report card is mainly concentrated within 2 min (accounting for 77.61%).

Analysis of efficiency improvement in filling out reporting cards. The Chinese Center for Disease Control and Prevention's infectious disease reporting cards contains 33 items. Before the system was launched, doctors mainly filled out the form using independent electronic spreadsheets, and patient information and addresses had to be obtained by asking the patient to enter the information. According to Wang et al.^39^, it took an average of 5 min for a doctor to complete a reporting form. After the system was launched, patient basic information, address, diagnosis, and other information were directly obtained through the system and electronic medical record system, and doctors only needed to verify and check the content related to infectious diseases, such as whether close contacts had the same symptoms after onset, disease classification, and so on. In order to verify the time saved by the system's automatic docking, the time when the doctor clicks the "report" button is used as the reporting time ${T}_{report}$, and the time when the doctor completes filling out the form and clicks the "submit" button is used as ${T}_{comfirm}$. Equation (3) is used to calculate ${T}_{2}$ to obtain the time it takes for doctors to fill out the reporting form. According to Fig. 11, 31.03% of doctors can complete the form in less than 1 min, 46.57% can complete it in 1–2 min, and less than 5% of doctors take more than 5 min, mainly owing to interruptions from seeing patients or answering phone calls while filling out the form, causing the page to stay on the reporting interface, resulting in the reporting time limit getting exceeded. Overall, by automatically filling in information, the average reporting time for each form filled out by a doctor has been reduced from an average of 5 min to less than 2 min, significantly improving the efficiency of doctors' work.

3 $${T}_{comfirm}-{T}_{report}={T}_{2}.$$

### Using interrupted time series to reduce underreporting rate analysis

In the past, the management of infectious disease reporting mainly relied on manual report verification^40^, and the use of clearly diagnosed report analysis in electronic medical records could remind doctors to report within the prescribed time limit in a timely manner. The main causes of missed reports that occur after the deadline are: (1) delayed laboratory test results; in clinical practice, many test results, such as bacterial culture and syphilis antibody testing, usually take 3–5 days until the results are ready, and in some cases, it may take several weeks for bacterial cultures to yield positive results, which cannot be promptly fed back to doctors; (2) patients transferred from outpatient or emergency departments to hospitals, as doctors usually focus on the primary symptoms of patients for treatment and may neglect to simultaneously check for infectious diseases; (3) infectious diseases such as hepatitis B and tuberculosis can be detected at hospital medical examination centers, but as the time required to issue medical examination reports is longer than that for normal outpatient and emergency departments, there is a risk of missed reports. According to Eq. (4), first given in Chen et al.^41^, the underreporting rate ${Underreport}_{0}$ is calculated as follows:

4 $$ \frac{Number\,of\,under - reported\,cases}{{Number\,of\,infectious\,disease\,cases\,in\,the\,same\,period}} \times 100\% = Underreport_{0} . $$

**Figure 12:**
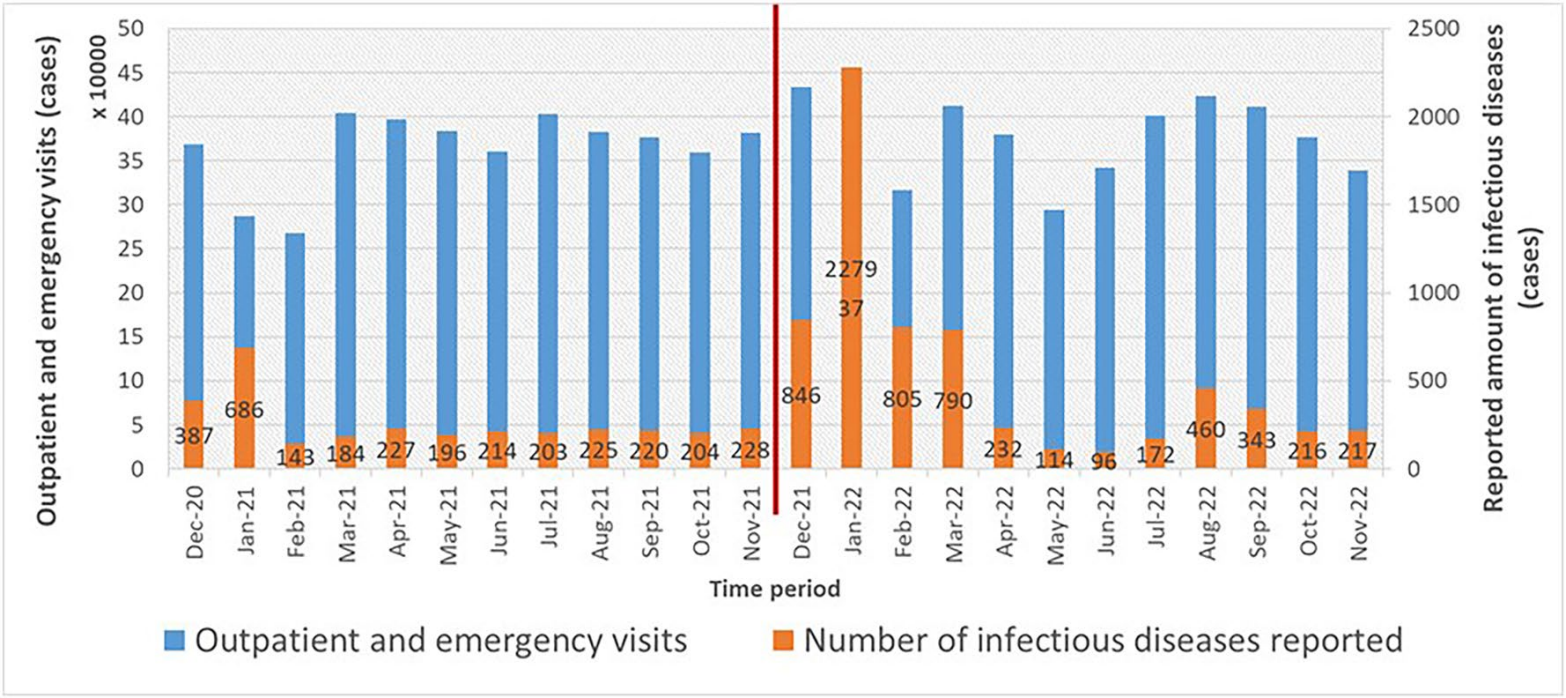
Outpatient and emergency volume and the number of reported infectious diseases before and after the system went online, with a red dividing line indicating the comparison before and after the launch.

Figure 12 shows the trend relationship between the number of hospital outpatient and emergency visits and the number of infectious disease reports from December 2020 to November 2022. Except for a noticeable increase in January 2022, the number of infectious disease reports remained relatively stable in other months. The p-value of the KS test for the underreporting rate data is 0.261 (no significant difference from the normal distribution), indicating that the data follow a normal distribution.

The total underreporting rate in 2021 is 179/3117 = 5.74%, and the total underreporting rate in 2022 is 80/6570 = 1.22%.

The Interrupted Time Series (ITS) method is used to evaluate the effectiveness of the reduction in under-reporting rates after system implementation by analyzing the change in slope and immediate level change at the intervention point. The data and parameters are presented in Table 4. Let Y represent the under-reporting rate and ${X}_{1}$ be the time variable for counting, with ${X}_{1}$ = 1, 2, 3…n. ${X}_{2}$ indicates the intervention, with ${X}_{2}$ = 0 before the intervention (December 2020 to November 2021), and ${X}_{2}$ = 1 after the intervention (December 2021 to November 2022). ${X}_{3}$ = 0 indicates observations before the intervention, and ${X}_{3}$ = ${X}_{1}$ indicates observations after the intervention.

1. Underreporting rate before and after the intervention: The average underreporting rate before the intervention was 5.7950%, the average under-reporting rate after the intervention was 1.3913%, and the average under-reporting rate after the intervention was 4.4037% lower than that before the intervention.
2. Level change model:

**Table 4:** Monthly underreporting rate and parameters after and before the system went online.

Time period	Number of infectious diseases reported	Number of underreported cases	Underreport_0_	${X}_{1}$	${X}_{2}$	${X}_{3}$
December 2020	387	21	5.43	1	0	0
January 2021	686	38	5.54	2	0	0
February 2021	143	8	5.59	3	0	0
March 2021	184	10	5.43	4	0	0
April 2021	227	13	5.73	5	0	0
May 2021	196	12	6.12	6	0	0
June 2021	214	13	6.07	7	0	0
July 2021	203	12	5.91	8	0	0
August 2021	225	13	5.78	9	0	0
September 2021	220	13	5.91	10	0	0
October 2021	204	12	5.88	11	0	0
November 2021	228	14	6.14	12	0	0
December 2021	846	12	2.08	13	1	13
January 2022	2279	24	1.72	14	1	14
February 2022	805	9	1.75	15	1	15
March 2022	790	9	1.05	16	1	16
April 2022	232	4	1.12	17	1	17
May 2022	114	2	1.14	18	1	18
June 2022	96	2	1.42	19	1	19
July 2022	172	2	1.16	20	1	20
August 2022	460	6	1.30	21	1	21
September 2022	343	4	1.17	22	1	22
October 2022	216	3	1.39	23	1	23
November 2022	217	3	1.38	24	1	24

Using underreporting rate as the dependent variable and ${X}_{2}$ as the independent variable, a simple linear regression model is fit:

$$\widehat{Y}={\widehat{\upbeta }}_{0}+{\widehat{\beta }}_{2}{X}_{2}=5.7950-4.4037{X}_{2}.$$

When ${X}_{2}$=0, the predicted missed report rate before intervention is:

$$\widehat{Y}={\widehat{\upbeta }}_{0}+{\widehat{\beta }}_{2}{X}_{2}=5.7950.$$

When ${X}_{2}$=1, the predicted missed report rate after intervention is:

$$\widehat{Y}={\widehat{\upbeta }}_{0}+{\widehat{\beta }}_{2}{X}_{2}=1.3913.$$

**Figure 13:**
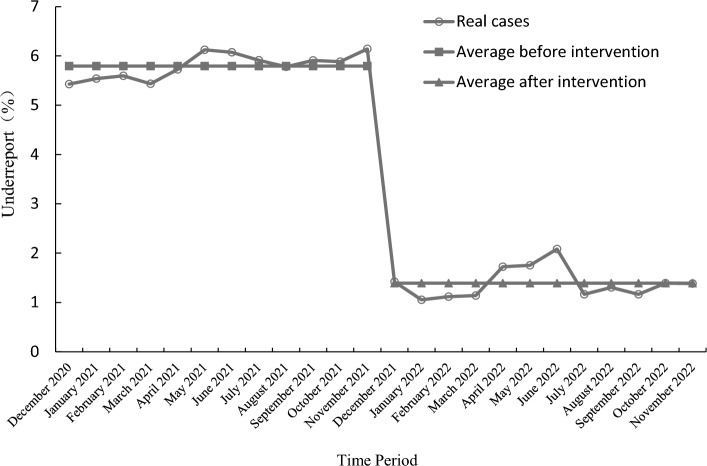
Change model of the level of underreporting rate.

The regression coefficient − 4.4037 indicates the impact of the intervention on the level of missed report rate, and as the coefficient is negative, the missed report rate after intervention is actually reduced by 4.4037 percentage points compared to before intervention. The p-value of the t-test for the regression coefficient ${\widehat{\beta }}_{2}$ is 0, < 0.01, indicating that the difference is statistically significant, as shown in Fig. 13.

(3) Level and slope change model:

The level and slope change model are fit as per Eq. (5):

5 $$Y={\beta }_{0}+{\beta }_{1}{X}_{1}+{\beta }_{2}{X}_{2}+{\beta }_{3}{X}_{3}+\epsilon .$$

Variables ${X}_{1}$,${X}_{2}$,${X}_{3}$ are substituted into Eq. (5). Before the experiment: ${X}_{1}$=1, 2…n, ${X}_{2}$ = ${X}_{3}$ = 0, then the model is:

$$Y={\beta }_{0}+{\beta }_{1}{X}_{1}+\upepsilon $$

After the intervention: ${X}_{2}$ = 1, ${X}_{3}$ = ${X}_{1}$, then the model is:

$$ Y = \beta_{0} + \beta_{1} X_{1} + \beta_{2} X_{2} + \beta_{3} X_{3} + \varepsilon_{t} = \beta_{0} + \beta_{1} X_{1} + \beta_{2} \times 1 + \beta_{3} X_{1} + \varepsilon = \beta_{0} + \beta_{2} + \beta_{1} + \beta_{3} X_{1} + \varepsilon_{t} = \beta_{0}^{*} + \beta_{1}^{*} X_{1} + \varepsilon . $$

${\beta }_{1}$ is the slope before intervention, ${\beta }_{2}$ is the level change, ${\beta }_{3}$ is the slope change, ${\beta }_{1}^{*}={\beta }_{1}+{\beta }_{3}$ is the slope after intervention, and ${\beta }_{0}^{*}={\beta }_{0}+{\beta }_{2}$ is the intercept after intervention.

Using data from Table 4 and fitting the multiple linear regression, the level and slope change model is obtained as:

$$\widehat{y}={\widehat{\upbeta }}_{0}+{\widehat{\beta }}_{1}{X}_{1}+{\widehat{\beta }}_{2}{X}_{2}+{\widehat{\beta }}_{3}{X}_{3}=5.4453+0.0537{\mathrm{X}}_{1}-3.2092{X}_{2}-0.0994{X}_{3}.$$

**Figure 14:**
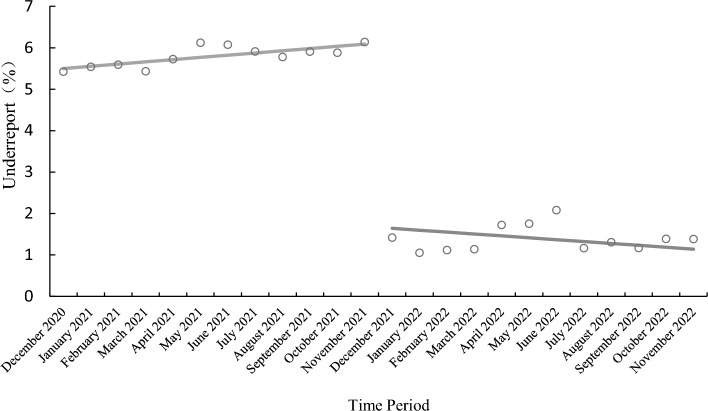
Model of the level and slope change of the missed report rate.

The slope before intervention is ${\widehat{\beta }}_{1}=0.0537$, and the slope after intervention is 0.0537 − 0.0994 =  − 0.0453. The slope change is ${\widehat{\beta }}_{3}$ = − 0.0994. The p-values of the t-test for ${\widehat{\beta }}_{1}$,${\widehat{\beta }}_{2}$, and ${\widehat{\beta }}_{3}$ are 0, 0.0119, and 0.0017, respectively, indicating that there is a significant change in the slope before and after intervention, as shown in Fig. 14.

## Discussion

### Real case generation association atlas improves the integrity and applicability of rules

Previous studies^42,43^ relied solely on neural network models for early warning prediction and diagnostic recommendations, making it difficult for doctors to rely on AI diagnostic recommendations in real clinical settings. The knowledge- and graph-based rule monitoring proposed in this study provides intuitive warning criteria and evidence-based decision-making support. Knowledge-based decision support does not simply digitize guidelines; rather, it requires retrospective research to continuously optimize the rules. The use of graphs further enhances and visualizes the rules summarized based on clinical guideline knowledge, facilitating rule modification that conforms to doctors' writing habits and extracts symptom keywords. After the system's launch in December 2021, the rules were continuously optimized. For instance, the rules related to influenza involved symptoms such as "fever," "cough," "headache," and "sore throat". However, these symptoms can be expressed differently, and doctors may have various writing habits. Therefore, it was necessary to standardize and unify the synonyms of symptoms. To achieve synonym transformation, the specific method involved converting the words into word vectors, thereby mapping semantically similar words to vectors with close proximity in vector space. This process was achieved by calculating the cosine similarity between the word vector of the target word and all standard word vectors. By finding the standard word vector with the closest distance, the target word was replaced with the corresponding standard word vector, completing the synonym transformation. In addition, in order to protect the privacy of infectious disease patients, doctors usually diagnose "urinary tract infection" rather than "genital chlamydia trachomatis infection," so relevant content is added to the monitoring rules. Based on the infectious disease guidelines and knowledge graph, the rules for assisted warning have authoritative guidance, are supplemented by graph symptoms based on real medical records, meet the hospital's personalized decision support needs, and improve the accuracy of early warning while reducing the possibility of symptom omission.

### Necessity of establishing a rule update and maintenance team

The rules in the system are dynamic and require timely updates as clinical guidelines evolve and new treatment options emerge. The rule update and maintenance team includes the following roles:

Medical Experts: Senior clinical experts with advanced titles are responsible for providing updates on the latest clinical guidelines and treatment protocols.

Information Engineers: They assist medical experts in configuring the rules within the system, which primarily involves the configuration of data field names.

Review Experts: They are responsible for reviewing the modified content, working closely with medical experts to address any potential conflicts.

Quality Management Experts: They monitor the updated rules. If a rule is marked as invalid more than 50% of the time within three months, they promptly provide feedback to Medical Experts and Review Experts for rule revision.

The collaboration among these team members ensures the continuous accuracy and relevance of the rules in the system, aligning them with the latest medical knowledge and best practices.

### The knowledge-based monitoring model proposed in this study can be extended to monitor other diseases

**Figure 15:**
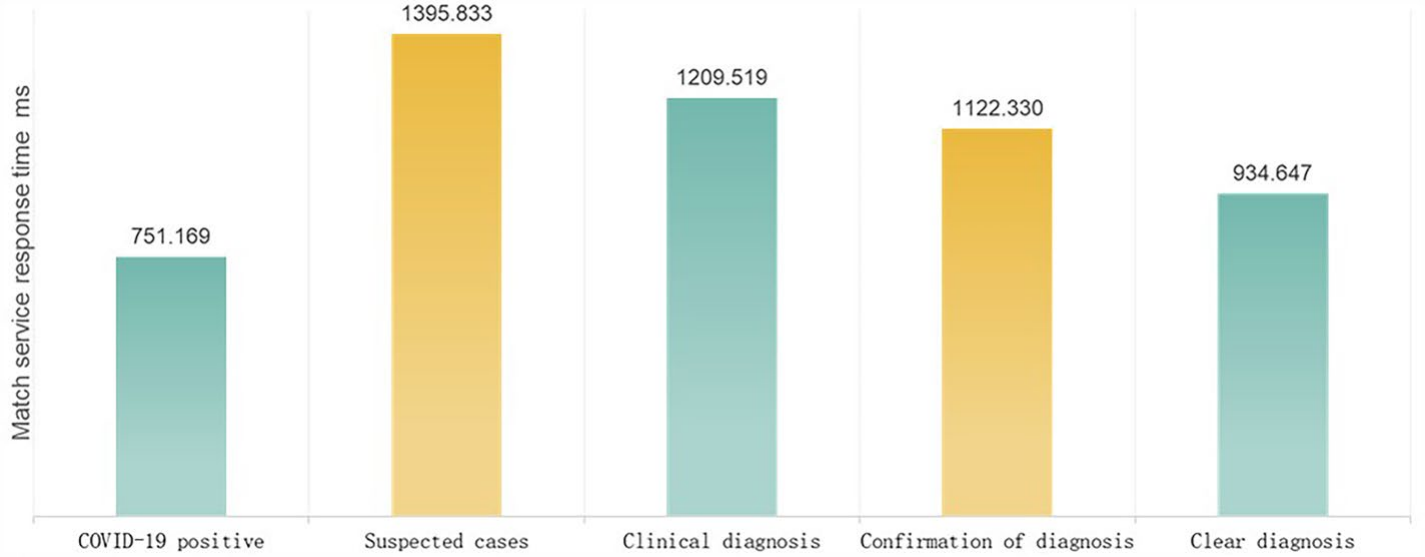
Response efficiency analysis of real-time monitoring service.

The comprehensive medical record monitoring method provides effective clinical decision support for non-communicable disease departments, such as the unexplained acute hepatitis in children that occurred in multiple countries in 2022^44^. The system provides pediatricians with early warning of viral hepatitis patients, enabling them to take relevant protective measures in advance. This study analyzed the efficiency of the system's real-time monitoring service response through log monitoring. As shown in Fig. 15, because suspected diagnosis, clinical diagnosis, and confirmed diagnosis all involve word segmentation and matching of medical records, the response time is slightly longer than that of direct diagnosis and positive test calls, but it can still respond within approximately 1 s, meeting the hospital's requirement for real-time disease warning monitoring. In addition, this model can be expanded to monitor other diseases based on rule content, such as foodborne disease monitoring, which will help reduce the incidence of diseases, timely detect outbreaks of microbial foodborne diseases, and quickly respond to control the spread of microbial foodborne diseases^41,45^. By configuring diagnoses of foodborne diseases such as "pesticide poisoning, pufferfish poisoning, mushroom poisoning, alcohol poisoning, and vibrio parahaemolyticus disease" as monitoring rules, and relevant test rules such as "bacterial culture, [{"test item": "bacterial culture"} and {"qualitative test result": "vibrio parahaemolyticus"}], it can signal the alert as "foodborne monitoring positive."

### Limitations

Considering that this study mainly targets only a few specific medical institutions in Beijing, China, where the incidence rates of some infectious diseases are low, the rules for infectious diseases that are affected by environmental exposure, such as echinococcosis, cannot currently be verified for their degree of conformity with actual situations. In addition, as the system adopts the MQ message mechanism^46^ to receive diagnostic data, there may be data transmission failures during peak data periods, which may cause the system to fail to monitor and issue warnings. When applying this system to other electronic health record systems, the differences between various medical institutions must be taken into account. For instance, there may be variations in data formats and programming languages among these systems. Therefore, some modifications are required to ensure seamless integration and compatibility with the diverse systems used in various medical institutions.

## Results

This study proposes a knowledge-based infectious disease monitoring and decision support system and validates its effectiveness and reliability. The system can accurately trigger valid alerts and has a low rate of false alarms, improving the infectious disease intelligent decision support for clinical doctors and enhancing the monitoring and management efficiency of disease control departments. In the event of a resurgence of infectious diseases, the rule update team can maintain the rule library based on the latest guidelines. Through the infectious disease monitoring and decision support system, doctors can receive assistance in determining whether a case is infectious, facilitating rapid diagnosis reporting. For hospital management departments, the system's increased reporting capabilities are complemented by a trend prediction model based on our previous research^4^, which enables early warning of outbreaks, thereby enhancing epidemic response capabilities and timeliness.

## Data Availability

The data that support the findings of this study are available from the corresponding author upon reasonable request.
